# Reorganisation of the Army Medical Service

**Published:** 1901-10-05

**Authors:** 


### Reorganisation of the Army Medical Service.

The report of Mr. Brodrick's Committee on the Reorganisation of the Army Medical Service, which has just been issued, shows that an earnest endeavour has been made to render the Medical Department attractive to young men leaving the medical schools. Whether there is any real intention of so reorganising the relations between the Royal Army Medical Corps and the combatant branches of the Army as to ensure that all medical and sanitary knowledge shall be brought into effectual use for the protection of the health of the troops in the field, is another question as to which the Report does not say anything. The first portion of the Report deals with the appointment of an Advisory Board. Of this Board the Director-General will be the Chairman, and it will contain representatives of the Royal Army Medical Corps, the War Office, and the India Office, together with two civilian physicians and two civilian surgeons, and (in regard to nursing matters only) the Matron-in-Chief, Queen Alexandra's Imperial Military Nursing Service. On this Advisory Board will be thrown a large proportion of the duties which have hitherto fallen upon the Director-General alone. Some may say, perhaps, that in undertaking these duties the Advisory Board will seriously undermine the position of the Director-General. On the other hand, however, it will give continuity in many matters in regard to which it is eminently undesirable that change of policy should follow change of *personnel*, and we can have no doubt that the Board will very usefully bring much special knowledge to bear upon questions regarding which no man who, by dint of years and much service, has attained the position of Director-General can be expected to speak with authority.

Then the Report goes on to lay down regulations for admission to the service and for the promotion and retirement of its officers. The first point to note is that the entrance examination will deal with professional subjects only. No one can doubt that many good men have been deterred from thinking of the Army as a career by the fact that for admission to Netley it was necessary to go back to elementary subjects and be examined in anatomy, physiology, chemistry, and pharmacy. All this is now done away with, and after undergoing his physical examination all the candidate will have to pass will be a clinical and practical examination in medicine and surgery, and as these are subjects in which a newly qualified man has just passed, such a test is not likely to deter any good man from entering the service. The very important proviso is made that any candidate who may pass the entrance examination while holding a resident appointment in a recognised civil hospital shall be seconded for the period of such appointment (not exceeding one year), receiving, however, no pay from Army funds, but counting his service towards pension or gratuity. This is a provision which will certainly be attractive to the very class the Army most wants—namely, house surgeons. On passing the entrance examination the candidate is appointed lieutenant on probation, and goes to Netley for two months, and then to Aldershot for three months for instruction in special duties, and is then examined in his work. If he passes he at once becomes lieutenant, and is attached to a battalion or other unit, doing duty at the same time at a station hospital. At the end of three years, during which he will be drawing pay at the rate (including allowances) of £323 10s. per annum, he will be permitted to retire, and we cannot but think that there are very many newly qualified men to whom the opportunity of passing three years with a regiment in this way will be a great attraction, even though they may not at first decide to permanently attach themselves to the service. If the Advisory Board consider that his service has been satisfactory he may either retire into the Reserve for seven years, receiving £25 per annum while so serving, or he may continue in the service. In the latter case he will be attached for six months, for purposes of study, to a recognised hospital, and then will undergo another examination. If he fail twice he must retire, but if he passes he then is promoted to the rank of captain. In this examination we find an arrangement by which, in proportion as men obtain marks in excess of the mere "pass" standard they will receive an acceleration of promotion, those who get 85 per cent. of the total marks receiving as much as 18 months of acceleration of promotion. This is very good, and as the same course is repeated in the examination for the rank of major, those who are clever at passing examinations have the chance of gaining three years of seniority towards gratuity or pension. On the completion of six years as captain, *i.e.* after nine years' service, an officer may retire with a gratuity of £1,000. If, however, he elects to remain, sometime between his ninth and twelfth year of total service he will be attached for six months to a hospital, after which he will be again examined. If he passes he then will be eligible for promotion to the rank of major; but if he fail twice he must retire. This examination covers a number of special subjects, such as skiagraphy, fevers, and even midwifery and pædiatrics, and a good pass in these subjects may lead to certain specialist appointments with additional "specialist pay" of 2s. 6d. a day. After 12 years of service an officer becomes major, and after being major for six years, that is, after 18 years of service (subject to the accelerations due to high place in examinations), he may either retire on a gratuity of £2,500 or continue in the service. If the latter, before being 20 years in the service, he will again have study leave for three months and pass another examination. If he fail after two tries at this examination he will be compulsorily retired with his gratuity of £2,500, or by special permission he may be allowed to complete his 20 years of service and retire on a pension. If he passes he will be eligible for promotion. But all further promotion is by selection, and failing promotion he must continue as major until he completes his 25 years of service, when he will be compulsorily retired on his pension. The Report then goes on to recommend some very desirable alterations in regard to the hospital arrangements and other matters. There are many points in the Report which will be strongly criticised from different quarters; but, looking at it from a recruiting point of view, we cannot doubt that it offers such attractions to newly qualified men as are likely to draw to the service a considerable number of medical officers of good position—and that is the first essential.

